# Effects of a Microbial Restoration Substrate on Plant Growth and Rhizosphere Microbial Community in a Continuous Cropping Poplar

**DOI:** 10.3390/microorganisms11020486

**Published:** 2023-02-15

**Authors:** Junkang Sui, Jiayi Yang, Chenyu Li, Lingxiao Zhang, Xuewen Hua

**Affiliations:** College of Agronomy and Agricultural Engineering, Liaocheng University, Liaocheng 252000, China

**Keywords:** poplar, rhizosphere, continuous cropping obstacle, microbial community, *Bacillus cereus*

## Abstract

In poplar cultivation, continuous cropping obstacles affect wood yield and soil-borne diseases, primarily due to structural changes in microbes and fungus infection. The bacterium *Bacillus cereus* BJS-1-3 has strong antagonistic properties against pathogens that were isolated from the rhizosphere soil of poplars. Poplar rhizospheres were investigated for the effects of *Bacillus cereus* BJS-1-3 on microbial communities. Three successive generations of soil were used to replant poplar seedlings. BJS-1-3 inoculated poplars were larger, had higher plant height and breast height diameter, and had a greater number of total and culturable bacteria than non-inoculated controls. *B. cereus* BJS-1-3 inoculated poplar rhizospheres were sequenced, utilizing the Illumina MiSeq platform to analyze changes in diversity and structure. The fungi abundance and diversity in the BJS-1-3 rhizosphere were significantly lower than in the control rhizosphere. In comparison to the control group, *Bacillus* sp. constituted 2.87% and 2.38% of the total bacterial community, while *Rhizoctonia* sp. constituted 2.06% and 6.00% of the total fungal community. Among the potential benefits of *B. cereus* BJS-1-3 in poplar cultivation is that it enhances rhizosphere microbial community structure and facilitates the growth of trees.

## 1. Introduction

A variety of cultivated tree species and poplars contribute to the ecology and economy of plantations through their use [1]. Globally, 8.6 million hectares of rapidly growing poplars are planted for the purposes of producing timber and environmental protection [2]. Poplar is one of the most widely planted tree species in the world. It has the advantages of fast growth, strong adaptability, high yield, good quality, wide varieties, easy hybridization, easy reproduction, and easy regeneration. It is a good material for artificial afforestation. In addition to providing paper, timber, and other wood-based products to the world, Populus also contributes to environmental protection [3]. However, continuous cropping for a long period of time has caused soil-borne diseases to flourish [4]. Under the long-term intensive continuous cropping management mode, the decline of plantation capacity is inevitable. Continuing cropping generally decreases soil nutrients, resulting in lower growth and greater incidences of Fusarium wilt disease (FWD) [5]. Furthermore, continuous cropping causes soil to change from bacterial dominant to dominant fungal type, as it promotes fungi growth and suppresses microbial growth [6].

Several causes might generate cropping obstacles, including soil erosion, soil-borne diseases, a discrepancy in nutrients, soil microbial community alterations, and autotoxic substance accumulation [7]. Long-term continuous cropping has been proven to affect soil microbial ecology. The modulation of soil microorganisms provides a way to deal with challenges associated with continuous cropping since these organisms play a crucial role in plant health and crop yield [8]. Plants in natural systems are constantly attacked by pathogens while being protected by a diversity of beneficial organisms [9]. For tomato plants in China, continuous cropping has resulted in serious soil-borne diseases [10]. Recent plantation practices, such as long-term continuous cropping of tomatoes (Lycopersicon esculentum Mill.), have caused microecological imbalances [11]. A long-term continuous cropping cycle results in a decrease in soil carbon, decreasing soil nitrogen and reducing soil nutrient availability [12]. A malfunction or deterioration in rhizosphere microorganisms is the primary cause of continual cropping difficulties [13]. Under continuous cropping, rhizobacterial soil communities must be understood to facilitate soil improvement [11].

In the soil, the rhizosphere environment contains potentially beneficial rhizobacteria that are found at the root–soil interface [14]. There are multiple endophytic species that originate from the rhizosphere, where root exudates and rhizodeposits attract microorganisms [15]. In some studies, local microbes found in the rhizosphere contribute to pathogen outbreaks and change the environment in the rhizosphere from healthy to diseased [16]. There has been substantial evidence that inoculating continuous cropping soil with beneficial microorganisms can overcome continuous cropping obstacles [17].

It has been well documented that Bacillus strains inhabiting the rhizosphere promote plant growth [18]. Plant-associated *B. amyloliquefaciens* strains colonize plant rhizospheres, promote plant growth, and suppress competing phytopathogens [19]. The plant-growth-promoting Bacillus strains can also stimulate the expression of expansins and ultimately contribute to plant growth promotion [20]. Additionally, Bacillus species produce auxins, which assist plant growth [21]. In particular, *Bacillus subtilis* and *Bacillus amyloliquefaciens* have a crucial function in the protection of plants from pathogens and plant growth induction [22]. *B. subtilis* biofilms have been linked to plant growth promotion [23]. It has been reported that in addition to promoting plant growth, *Bacillus amyloliquefaciens* FZB42 is also able to produce a large number of secondary metabolites that are non-ribosomal [24]. *Bacillus cereus* may potentially be used as a plant-growth-promoting bacterium [25], with the potential to synthesize plant-growth hormones [26].

Therefore, we performed a field experiment after three-generation continuous cropping of poplar. A bactericidal strain BJS-1-3 was utilized to study bacterial and fungal community structures among rhizospheres of continuously cropped poplars. Utilizing both growth of poplar trees and bacteria and fungi biomass, we evaluated whether BJS-1-3 could alleviate continuous cropping challenges and improve microbial structure.

## 2. Materials and Methods

### 2.1. Experimental Site and Design

The study location (31°56′ N, 117°08′ E) is in Taian City in Shandong Province, in eastern China. Despite its proximity to the Dawen River, it has not recently seen floods. This region is characterized by a climate that is classified as temperate monsoon, typically with 195 frost-free days annually. July–September has an average yearly temperature of 12.9 °C (from −20.7 °C to 38.1 °C) and 697 mm of precipitation.

Approximately 3.5 km^2^ of artificial forest was created for the experiment. Treatment areas were rectangular, 12 m by 80 m, running nearly from south to north. A block design was used in the artificial forest study. Across each treatment area, three parallel rows were planted with 2-meter-apart seedling rows that were four meters apart. A distance of 20 m separated the BJS-1-3 group from the control (CK) group.

The poplar seedlings were planted for three generations on this site. Following three generations of non-rotated cropping, on 30 March 2018, the same-height seedlings were replanted. The BJS-1-3 strain is deposited in the Microbiology Institute, Chinese Academy of Sciences, with CGMCC deposit number 6635. We injected seedlings with the BJS-1-3 zymotic fluid that contained 9.27 × 10^8^ cfu/mL microorganisms. Upon fermentation of BJS-1-3, the fermentation medium consisted of 50 g/L corn flour, 30 g/L corn syrup, 10 g/L glucose, 6 g/L NaCl, 2 g/L MnSO^4^, and distilled H_2_O. The roots were submerged in media for five minutes in the first treatment. As a seedling was grown, the fermentation media was diluted tenfold with irrigation water, which was then used to inoculate with 1.55 × 10^10^ cfu (1.55 × 10^6^ cfu/g soil) bacteria. As soon as the seeds were planted, 200 mL of diluted medium per seedling was used to irrigate the roots. Finally, 4 L of medium-diluted water was used to irrigate the seedlings. In the same way, sterile zymotic fluid was applied to the control group (CK). About 60 seedlings were treated per treatment. In October 2018 and October 2020, the DBH (1.3 m above ground) of every seedling was measured.

### 2.2. Collecting Samples and Counting Culturable Microorganisms

On 30 October 2020, the roots of five trees were randomly selected from the north, south, and central regions. We discarded extra bulk and referred to soil sticking to roots as rhizosphere soil (Smalla et al., 1992). Each location yielded a total of three triplicate soil samples, 15–20 cm deep, for each treatment, by mixing soil samples acquired at 15–20 cm depths in the south, center, and north. Soil samples were transported to our lab in sterile plastic bags, placed on ice, and kept at −80 °C until DNA isolation. To obtain final samples for analysis, replicate samples were pooled, then quartiles were used (BJS-1, BJS-2, BJS-3, and CK1, CK2, CK3).

We serially diluted soil samples up to 10-fold and counted the bacteria after inoculating them on nutrient agar medium. We also counted the fungi on Martin agar media with 30 g/mL streptomycin. At 28 ± 2 °C, agar plates were incubated for 2–3 days [27].

### 2.3. Edaphic Properties Determination

Soil organic carbon (OC) was assessed by the Walkley–Black method [28]. We mixed 10 mL of 1 N potassium dichromate with 20 mL of concentrated H2SO4, introduced 0.1 g of sieved and dried soil, and gently rotated the mixture for one minute. Incubation was performed at 150 °C for 10 min, and cooling was achieved at room temperature. As a next step, 200 mL of deionized water, 0.2 g NH_4_F, ten drops of (C_6_H_5_)_2_NH indicator, and 10 mL of H_3_PO_4_ were used to dilute samples to 200 mL. Titration of excess dichromate was then carried out with Mohr salt solution (0.5 N FeNH_4_SO_4_ and 0.1 N H_2_SO_4_). The molybdenum blue method was used to determine the availability of P (AP), and a spectrophotometer (UV2550, Tokyo, Japan) was used to evaluate the data [29]. After extracting the soil with C₂H₇NO₂, available K (AK) was determined. Quantification of Total N (TN) was performed utilizing an automatic Kjeldahl distillation-titration unit (Foss, Höganäs, Sweden). With the help of a pH meter, soil samples were measured for pH (Mettler Toledo, Greifensee, Switzerland).

### 2.4. DNA Isolation and PCR Amplification

Utilizing an E.Z.N.A. soil DNA isolation kit, we retrieved DNA from soil samples collected across two separate groups (Omega Bio-Tek, Norcross, GA, USA). The V3–V4 region of bacterial 16S ribosomal RNA was tested by PCR (95 °C, 3 min; and subsequently by 27 cycles of 95 °C for 30 s, 55 °C for 30 s, and 72 °C for 45 s; and a final extension at 72 °C, 10 min) utilizing primers as follows: 806R, 5′-GGACTACHVGGGTWTCTAAT-3′ and 338F, 5′-barcode-ACTCCTACGGGAGGCAGCA-3′ [30]. Barcode consists of an 8-nucleotide sequence unique to all samples. A 20 L reaction mixture was used for PCR in triplicate: 2.5 mM dNTPs (2 μL), 5× FastPfu Buffer (4 μL), each primer (5 μM; 0.8 μL), 10 ng template DNA, FastPfu Polymerase (0.4 μL), and ddH_2_O to 20 µL volume.

Conditions for fungal ITS1 PCR were 95 °C, 3 min; and subsequent 35 cycles at 95 °C for 30 s, 55 °C for 30 s, and 72 °C for 45 s; and at 72 °C final extensions, 10 min) utilizing primers as follows: ITS1F, 2043R, 5′-GCTGCGTTCTTCATCGATGC-3′ and 5′-barcode-CTTGGTCATTTAGAGGAAGTAA-3′ [31]. Barcode consists of an 8-nucleotide sequence unique to each sample. A 20 L reaction mixture was used for PCR in triplicate, as previously mentioned.

### 2.5. Real-Time (q)PCR

The oligonucleotide primers used for 16S ribosomal RNA V3–V4 regions amplification of bacteria and ITS1 regions of fungi are described in Section 2.3. The reaction mixture for qPCR contained each primer (10 μM; 0.5 μL), 25-μL of 2× SYBR Green qPCR Master Mix (12.5 μL), template DNA (2 μL) and ddH2O (9.5 μL). Melting-curve analysis and amplification conditions of 95 °C for 10 min, 40 cycles of 95 °C for 15 s, and 60 °C for 1 min. An ABI 7500 fluorescence quantitative analyzer was used for data analysis, with a baseline start of 3 and a baseline end of 11 to 12.

### 2.6. Illumina MiSeq Sequencing

An AxyPrep DNA Gel Extraction Kit was utilized to purify amplicons from 2% agarose gels (Axygen Biosciences, Union City, CA, USA), following the instructions of the manufacturer. Then, a QuantiFluor-ST Kit quantified the products (Cat No, Promega, Madison, WI, USA). A paired-end sequencing protocol on the Illumina MiSeq platform was used, using pooled amplicons in equimolar amounts following the instructions of the manufacturer.

### 2.7. Data Processing for Sequencing

Using the Mothur software uchime function, we removed chimera sequences and obtained high-quality sequences for additional analysis. Following raw fastq files de-multiplexing, QIIME (version 1.9.1), quality-filtering was performed as follows: (i) For mass values above 20 in the window, a 50 bp sliding window was applied, all sequences from the rear end of base to the front end of window were removed, sequences whose length exceeded 50 bp were removed after quality control. (ii) Overlap bases were spliced according to overlap sequences. The greatest mismatch rate was 0.2%, and the overlap exceeded 10 bp. Unstitchable sequences were removed. (iii) A barcode was used to separate the sequences in each sample, as well as primers at both ends. There was no tolerance for sequences including unclear bases (primers could have two mismatched bases) [32].

UPARSE V. 7.1 was applied to cluster OTUs (operational taxonomic units) with a 97% similarity threshold (http://drive5.com/uparse/; accessed on 19 March 2020) [33,34], and UCHIME was utilized to assess and remove Chimeric sequences [35]. Using an RDP Classifier (http://rdp.cme.msu.edu/; accessed on 19 March 2020) with a 70% confidence threshold, every 16S rRNA gene taxonomy was compared against Silva’s (SSU128) 16S rRNA data resource [36]. An RDP Classifier was utilized to evaluate each ITS sequence over UNITE 7.0/ITS data set at a 70% confidence level [37].

### 2.8. Statistical Analyses

The results were presented as mean + standard deviation (SD). An analysis of variance followed by a multiple ranger test with a significance level of *p* < 0.05 revealed noteworthy variations among culturable and total microorganisms, plant height, poplar trees-DBH, and diversity along with richness indices between BJS-1-3 and CK groups. SAS version 9 was used to perform all statistical analyses (SAS Institute Inc., Cary, NC, USA).

## 3. Results

### 3.1. DBH and Plant Height Changes in Poplar Trees

Compared with non-inoculated CK trees, the poplars treated with BJS-1-3 had 9.34% thicker trunks and 3.96% higher heights in October 2018. BJS-1-3-treated poplars were found to be 10.30% thicker and 5.03% higher than CK-treated poplars in October 2020. The DBH of BJS-1-3 group grew by 84.06 mm, and that of the CK group by 75.90 mm from October 2018 to October 2020 (Table 1). A 7.12 m height increase was observed in the BJS-1-3 group, while a 6.74 m height increase was observed in the CK group. The elevated DBH and plant-height values indicated that applicated BJS-1-3 strain promoted the growth of poplar BJS-1-3.

### 3.2. Variations in Edaphic Characteristics between March 2018 and October 2020

In March 2018, neither BJS-1-3 zymotic fluid nor sterile zymotic fluid (CK) significantly altered the OC, AK, AP, TN, or pH of the soil (Table 2). The OC in the BJS-1-3 treatment group was significantly greater than those among the CK group. Soil AP, AK, and TN concentrations were significantly greater than in CK plots in October 2020, at 8.58 (mg/kg), 47.09 (mg/kg), and 63.35 (mg/kg), respectively. The seedling group treated with BJS-1-3 showed a marked increase in soil pH. Comparing October 2020 to March 2018, CK plots showed significant increases in soil OC and AK. However, there was no significant change in either AP or AK between March 2018 and October 2020.

### 3.3. Microbial Quantities in Poplar Rhizosphere Soils

Unlike CK, BJS-1-3 fermentation broth greatly enhanced the number of culturable bacteria in rhizosphere soil. BJS-1-3 rhizospheres contained nearly ten times more culturable bacteria than CK rhizospheres. Despite this, 62.16% of the culturable fungi were found in BJS-1-3 rhizospheres compared to CK rhizospheres (Table 3). According to qPCR results, the BJS-1-3 rhizosphere contained 74.64% more bacteria than the CK rhizosphere but 29.83% fewer fungi than the CK rhizosphere (Table 3).

### 3.4. Estimates of Microbial Community Diversity and Species Richness

**Figure 1:**
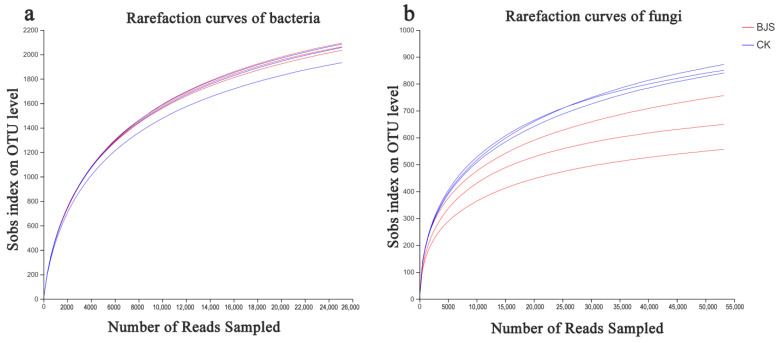
A rarefaction curve of bacterial communities (**a**) and a rarefaction curve of fungal communities (**b**) showing the influence of a 3% dissimilarity cutoff on uncovered OTUs. “BJS-1-3” means BJS-1-3 fermentation-broth-treated poplar group; “CK” means sterilized fermentation-broth-treated poplar group. For each treatment, three replicates were analyzed.

**Figure 2:**
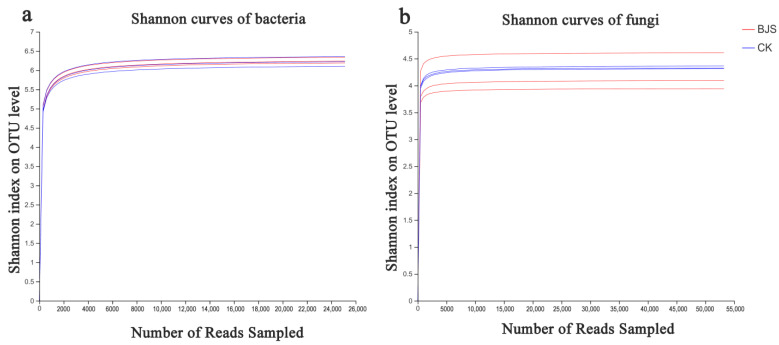
A Shannon curve of bacterial communities (**a**) and Shannon curves of fungal communities (**b**). Shannon curves illustrate the effects of a 3% dissimilarity cutoff on uncovered OTUs. “BJS-1-3” means BJS-1-3 fermentation-broth-treated poplar group; “CK” means sterilized fermentation-broth-treated poplar group. For each treatment, three replicates were analyzed.

Filtered sequencing data excluded short reads and low-quality reads, resulting in 47,669 and 50,480 bacterial 16S rDNA, respectively, and 72,637 and 68,753 fungal ITS sequences. Reads were classified into diverse OTUs, utilizing a clustering dissimilarity cutoff of 3%. Neither bacterial nor fungal 0.03 distance level rarefaction curves reached an asymptote (Figure 1). Thus, not all communities among samples were fully represented by sequencing data. As read counts increased, Shannon diversity curves plateaued (Figure 2), according to the integration of Shannon diversity index and rarefaction curves. As a result, to examine communities, sufficient data were collected.

Analysis was conducted on soil samples to determine their diversity and richness indices (Table 4). Notably, bacterial diversity and richness were similar between BJS-1-3 and CK groups. According to ACE (abundance-based coverage estimator) with Chao values, indicating species richness, the rhizosphere bacteria in the BJS-1-3 community were relatively rich. In addition, in contrast to BJS-1-3, the OTU values in CK were slightly lower. The diversity indices of Shannon and Simpson displayed a similar pattern; lower diversity indices were related to larger Simpson values.

A dramatic difference was observed between the BJS-1-3 group and CK group according to ACE and Chao values for fungi. Furthermore, the Shannon diversity index in the BJS-1-3 was significantly lower than that among the CK group. A lower diversity was indicated by a higher Simpson index in the BJS-1-3 group. As a result of these data, it was evident that fungal communities are richer and more diverse in the CK group than in the BJS-1-3 group. There were only minor differences between BJS-1-3 and CK regarding bacterial community diversity and species richness.

### 3.5. Composition and Structure of Communities Affected by BJS-1-3

**Figure 3:**
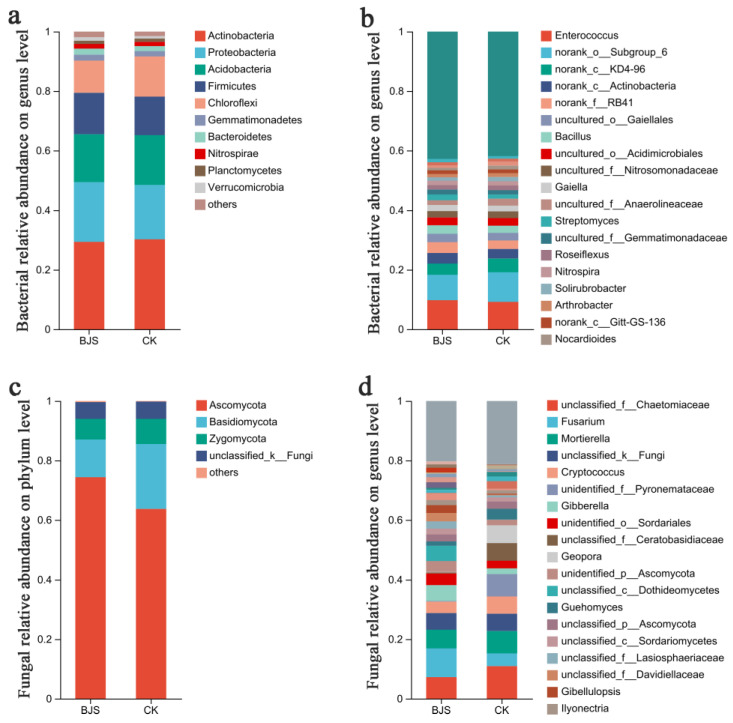
Bacterial and fungal communities in studied groups. (**a**) Bacterial relative abundance at phylum level; (**b**) Bacterial relative abundance at genus level; (**c**) Fungal relative abundance at phylum level; (**d**) Fungal relative abundance at genus level. An illustration of the relative abundances of major members in stacked bar graphs. “BJS” means BJS-1-3 fermentation-broth-treated poplar group; “CK” means sterilized fermentation-broth-treated poplar group. For each treatment, three replicates were analyzed.

Classification of the sequences was performed using the Mothur program. Actinobacteria, Proteobacteria, and Acidobacteria were dominant phyla among both groups; they were 29.34%, 20.06%, and 16.10%, respectively, in the BJS-1-3 group, and 30.21%, 18.29%, and 16.70%, respectively, in CK group. Ascomycota and Basidiomycota were dominant phyla for both groups; they were 74.42% and 12.60%, respectively, in the BJS-1-3 group and 63.76%, and 21.78%, respectively, in the CK group. Actinobacteria, Proteobacteria, and Acidobacteria were predominant bacterial phyla based on heatmap analysis. Ascomycota and Basidiomycota were the most predominant fungal phyla (Figure 3a,c).

**Figure 4:**
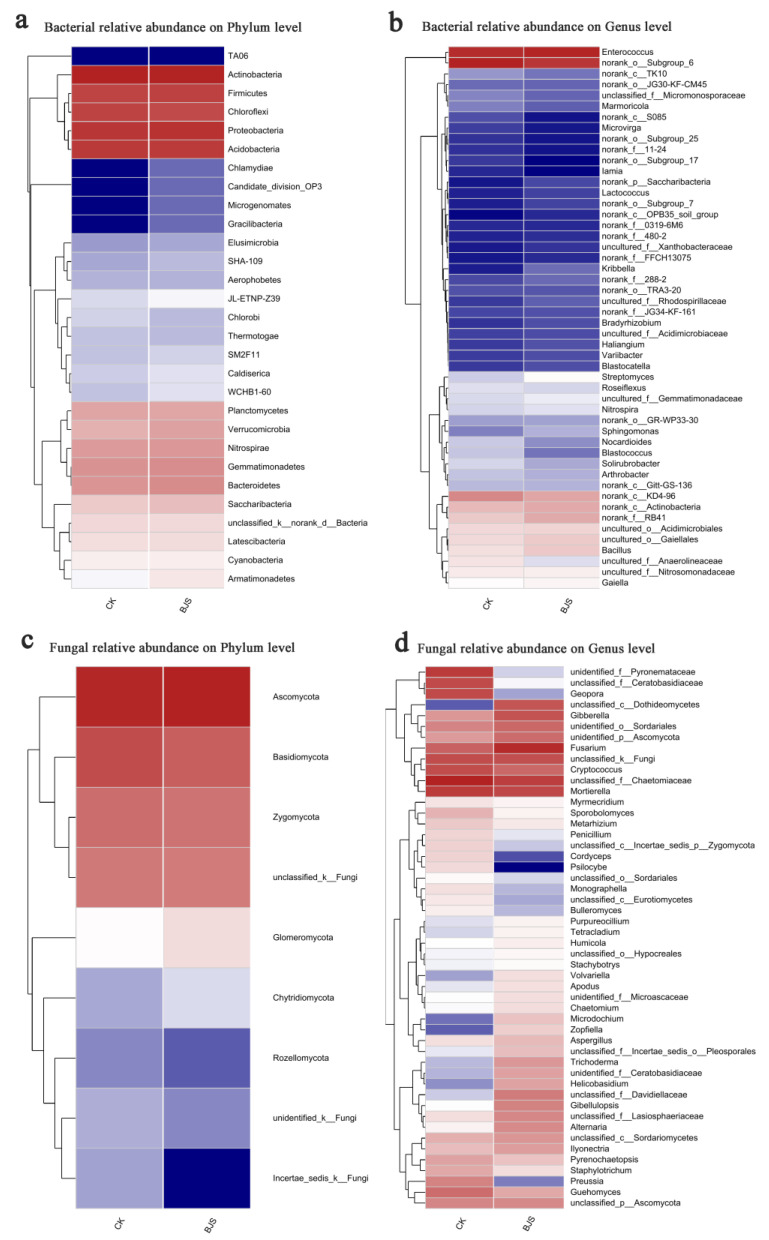
Hierarchical clustering heatmaps of bacteria and fungi. (**a**) Bacterial relative abundance at the phylum level; (**b**) Bacterial relative abundance at the genus level; (**c**) Fungal relative abundance at the phylum level; (**d**) Fungal relative abundance at the genus level. Communities within the two groups are visualized based on their genus in the heatmaps. “BJS” means BJS-1-3 fermentation-broth-treated poplar group; “CK” means sterilized fermentation-broth-treated poplar group. For each treatment, three replicates were analyzed.

Overall bacterial compositions of the two groups were similar, but genus distributions differed (Figure 3). There was 9.72% of classifiable bacterial sequences from Enterococcus in the BJS-1-3 group but 9.27% among CK group (Figure 3b). Accordingly, the *Bacillus* fractions in the BJS-1-3 group and CK group were 2.87% and 2.38%, respectively. BJS-1-3 had a *Nocardioides* proportion of 0.92%, which was lower than reported in CK group (1.37%). Similarly, A lower *Blastococcus* fraction (0.77%) was detected in the BJS-1-3 group as compared to the CK group (1.47%). BJS-1-3 had an *Arthrobacter* proportion of 1.13%, which was significantly lower than CK (1.69%). *Nitrospira* was found in 1.56% of the BJS-1-3 group, significantly higher than the CK group (1.13%). *Streptomyces* were present in 1.96% of the BJS-1-3 group, which was greater than CK group (1.37%). A remarkably higher percentage of *Gaiella* was found in the BJS-1-3 group (2.73%) than in the CK group (1.95%). In contrast, BJS-1-3 showed a remarkably lower percentage of Roseiflexus (1.45%) than CK (1.95%). According to the heatmap, there were also quantitative associations of bacteria and fungi in BJS-1-3 and CK. Enterococcus fractions were 15.11% and 10.6% in the BJS-1-3 and CK groups, respectively, representing the largest differences in bacteria. In contrast, *Blastococcus* formed 0.54% of the population, *Bacillus* sp. formed 3.44% and 2.74%, *Anaerobic rosaceae* formed 2.34% and 2.72%, respectively, in the BJS-1-3 and CK groups (Figure 4b).

The Ascomycota in the BJS-1-3 group accounted for 9.48% of total classifiable fungal sequences but only for 8.44% in the CK group (Figure 3d). In the BJS-1-3 and CK groups, Geopora fractions were 0.12% and 5.99%, respectively, BJS-1-3 had a *Fusarium* fraction of 9.81%, which was significantly higher than CK (4.03%). In comparison to the CK group, the BJS-1-3 group had a fraction of Rhizoctonia of 0.29%, which was significantly lower than that of the CK group (5.82%). Numerous diseases in plants are caused by *Fusarium* and *Rhizoctonia*. There was 2.83% of *Cryptococcus* in the BJS-1-3 group, which was lower than the CK group (4.93%). Furthermore, the BJS-1-3 group had a fraction of *Preussia* of 0.27%, which was significantly lower than the CK group (2.72%). As for fungi, heatmap analysis showed that BJS-1-3 and CK contained 10.19% and 6.17% *Geopora*, respectively. For BJS-1-3 and CK groups, Fusarium species accounted for 4.39% and 4.15%, while Rhizoctonia species accounted for 2.06% and 6.00%, respectively. (Figure 4d).

**Figure 5:**
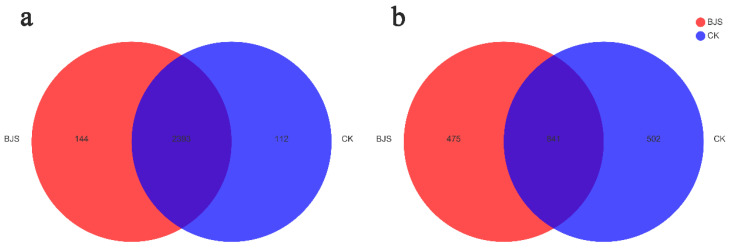
A Venn diagram illustrating the differences and similarities between the two treatments in terms of bacterial (**a**) and fungal (**b**) OTUs. “BJS” means BJS-1-3 fermentation-broth-treated poplar group; “CK” means sterilized fermentation-broth-treated poplar group. For each treatment, three replicates were analyzed.

As shown in Figure 5, both bacterial and fungal OTUs were prevalent in both study groups. At 3% dissimilarity, there were 2537 OTUs for BJS-1-3 and 2505 for CK. There are 2393 OTUs shared between the BJS-1-3 group and the CK group. It was found that *Enterococcus*, *Bacillus*, and *Acidothermus* were the most frequent genera in both groups. It is only in the BJS-1-3 group that *Chryseobacterium* and *Glycomycetaceae* were found. *Rhodocyclaceae*, *Moraxellaceae,* and *Oscillochloris* were only found in the CK group.

At the 3% dissimilarity, we found 1316 fungal OTUs in the BJS-1-3 group and 1343 in the CK group. There are 841 OTUs shared between the BJS-1-3 group and the CK group. It was found that *Mortierella*, *Fusarium*, *Cryptococcus*, *Geopora*, and *Cladosporium* were the most frequent genera in both groups. It is only in the BJS-1-3 group that *Chaetosphaeronema*, *Ganoderma*, *Dissoconium*, and *Endosporium* were found, while it is only in the CK group that *Archaeorhizomyces*, *Camarosporium*, *Elaphomyces*, and *Bionectria* were found.

## 4. Discussion

Planting crops in the same plot over many years can result in crop growth conditions deteriorating, yield and quality reductions, as well as disease aggravation [38]. A variety of factors have been identified as contributing to continuous cropping obstacles, such as soil-borne diseases, nutrient imbalances, and deterioration in soil physicochemical properties in soil bacterial and fungal communities [7]. It is estimated that continuous cropping obstacles result in severe mortality of plants [39]. Continuous cropping obstacles are mainly caused by the disruption of microorganisms in the rhizosphere [40]. Over time, continuous cropping has been shown to influence the microbial soil community, accelerating the abundance of different taxa in the soil community [41].

In comparison with the control group, the DBH of the BJS-1-3 treatment group increased by 11.45 mm on average 16 months after the treatments. The plant height data showed a 7.12 m increase in the BJS-1-3 group, larger than the 6.74 m in the CK group. These results indicate that BJS-1-3 can promote the production of poplar trees. Similarly, according to the report, *Bacillus cereus* YN917 has multiple growth-promoting characteristics that may make it suitable for use as a bio-fertilizer [42].

As a result of using strain BJS-1-3 in the rhizosphere of poplars, the number of bacteria, culturable and total, increased, and the number of fungi decreased dramatically. As a result of BJS-1-3 treatment, OC, AP, AK, and TN were also increased obviously. In this situation, soil dominated by fungi and having low fertility can be improved by the application of BJS-1-3. An earlier study suggested that when beneficial microbial populations decrease and pathogenic microorganisms increase in soil, the rhizosphere soil microflora and bacteria are replaced by fungi, which are less fertile [43]. As a result of prolonged continuous cropping, soil microbial community diversity would become less, and harmful microbial populations would increase [44]. As a result of the continuous cropping of alfalfa over a long period, soil properties changed, the structure of the soil fungal community changed, and the soil fungal alpha diversity increased [6].

The bacterial diversity and richness indices in the BJS-1-3 group and CK group are not obviously different. The dominant phyla were Actinobacteria, Proteobacteria, and Acidobacteria, and many studies have indicated their importance as antagonistic microorganisms in soils. In soil, for instance, Acidobacteria is ubiquitous [45]. In addition, studies have shown that Acidobacteria is involved in the degradation of plant and microorganism-derived polysaccharides and that they are correlated with soil N availability [46]. As a result of sequencing, it was shown that the majority of genus sequences identified were those of *Enterococcus*, the most abundant genus in the BJS-1-3 rhizosphere and also in the CK rhizosphere. A higher fraction of *Bacillus* was found in BJS-1-3 rhizospheres compared to CK rhizospheres, suggesting that *Bacillus* BJS-1-3 may improve the structure of the bacterial community in the rhizosphere. There was a higher fraction of *Rhizoctonia* in the CK plot compared to that in the BJS-1-3 plot in our study. This is consistent with previous studies [47,48].

Moreover, the most enriched phylum in both study samples was Ascomycota, which comprised the majority of the fungal sequences. A number of studies have demonstrated the importance of Ascomycota in soils and their ubiquitous presence [49,50]; it is also the dominant fungi in continuous cropping soil [51,52]. As compared to the CK rhizosphere, the BJS-1-3 had a significantly lower fraction of *Rhizoctonia*; the presence of *Rhizoctonia* pathogens may account for continuous cropping obstacles during poplar cultivation [53]. It has been known for a long time that *R. solani* is an important root pathogenic fungus among a variety of crops [54,55,56]. Various types of crops are affected by *Rhizoctonia solani*, a major root pathogen that is helpful in developing natural disease suppression under continuous cropping situations [57,58].

By applying *B. cereus* BJS-1-3 fermentation broth, we were able to promote poplar production, inhibit pathogenic fungi in the soil, and enhance microbial community structure in the rhizosphere. In addition, researchers have found that *B. cereus* could be used as an environmentally compatible plant-growth-promoting and pathogen residence agent. For example, Bacillus cereus MH778713 could be used as a biocontrol agent to control *Fusarium*-induced plant diseases [59]. Similarly, the plant-growth-promoting bacteria *Bacillus cereus* YN917 has remarkable antifungal activity [42].

## 5. Conclusions

As a result, we demonstrated that *B. cereus* BJS-1-3 can go a long way toward alleviating the constraints of continuous poplar cropping. Poplar trees continuously cropped with this product grew faster, had a more diverse rhizosphere microbe community, and were less susceptible to pathogenic fungi. BJS-1-3 has the potential ability to be used as a biocontrol agent strain to protect poplar trees against factors that limit poplar cultivation during continuous cropping.

## Figures and Tables

**Table 1:** DBH and plant height changes in poplar trees.

Treatment	DBH (mm) in 2018/10	DBH (mm) in 2020/10	Plant Height (m) in 2018/10	Plant Height (m) in 2020/10
BJS-1-3	38.52 ± 4.13 ^a^	122.58 ± 8.73 ^a^	4.15 ± 0.28 ^a^	11.27 ± 0.56 ^a^
CK	35.23 ± 4.12 ^b^	111.13 ± 8.84 ^b^	3.99 ± 0.30 ^a^	10.73 ± 0.48 ^b^

Data are presented as mean ± SE. Different lowercase superscript letters indicate notable differences at *p* < 0.05. “BJS-1-3” means BJS-1-3 fermentation-broth-treated poplar group; “CK” means sterilized fermentation-broth-treated poplar group.

**Table 2:** Soil edaphic characteristics between March 2018 and October 2020.

		OC (g/kg)	AP (mg/kg)	AK (mg/kg)	TN (mg/kg)	pH
March 2018	BJS-1-3	6.06 ± 0.05 ^c^	46.23 ± 0.13 ^b^	131.44 ± 0.82 ^b^	771.78 ± 3.26 ^b^	7.21 ± 0.01 ^b^
	CK	6.05 ± 0.07 ^c^	35.63 ± 2.33 ^b^	132.28 ± 1.44 ^b^	765.82 ± 7.43 ^b^	7.23 ± 0.02 ^b^
October 2020	BJS-1-3	7.14 ± 0.02 ^a^	43.96 ± 0.70 ^a^	186.29 ± 2.40 ^a^	854.93 ± 3.94 ^a^	7.40 ± 0.01 ^a^
	CK	6.27 ± 0.04 ^b^	35.38 ± 2.77 ^b^	139.20 ± 2.03 ^b^	791.58 ± 3.77 ^b^	7.07 ± 0.04 ^c^

Data are presented as mean ± SE. Different lowercase superscript letters indicate notable differences at *p* < 0.05. The dates “March 2018” and “October 2020” indicate the days before and after zymotic fluid and sterile zymotic fluid treatments; “BJS-1-3” means BJS-1-3 fermentation-broth-treated poplar group; “CK” means sterilized fermentation-broth-treated poplar group.

**Table 3:** Microbial quantity in rhizosphere soils of both treatments.

Treatment	Culturable Microbial Contents after the Two Treatments		Total Microbial Contents after the Two Treatments	
	Bacterial × 10^7^ (cfu/g Soil)	Fungal × 10^6^ (cfu/g Soil)	Bacterial × 10^7^ (Copies/μL)	Fungal × 10^4^ (Copies/μL)
BJS-1-3	9.56 ± 0.21 ^a^	5.75 ± 0.31 ^b^	1.24 ± 0.23 ^a^	2.07 ± 0.08 ^b^
CK	0.96 ± 0.17 ^b^	9.25 ± 0.49 ^a^	0.71 ± 0.02 ^b^	2.95 ± 0.10 ^a^

Data are presented as mean ± SE. Different lowercase superscript letters indicate notable differences at *p* < 0.05. “BJS-1-3” means BJS-1-3 fermentation-broth-treated poplar group; “CK” means sterilized fermentation-broth-treated poplar group.

**Table 4:** Diversity along with richness indices of bacterial, as well as fungal communities from the two soil treatments.

	Sample	Cutoff	OTUs	ACE	Chao	Shannon	Simpson	Coverage
Bacterial	BJS-1-3	0.03	2209 ± 51.36 ^a^	2491.02 ± 62.03 ^a^	2451.81 ± 88.31 ^a^	6.24 ± 0.06 ^a^	0.011 ± 0.003 ^a^	0.980661
	CK	0.03	2106 ± 106.71 ^a^	2384.41 ± 82.60 ^a^	2402.16 ± 78.39 ^a^	6.20 ± 0.12 ^a^	0.012 ± 0.004 ^a^	0.980821
Fungal	BJS-1-3	0.03	793 ± 68.93 ^a^	775.30 ± 50.50 ^b^	793.31 ± 63.1 ^b^	3.97 ± 0.30 ^b^	0.057 ± 0.019 ^a^	0.997483
	CK	0.03	901 ± 7.55 ^a^	1032.86 ± 57.48 ^a^	1022.94 ± 93.32 ^a^	4.34 ± 0.02 ^a^	0.040 ± 0.005 ^b^	0.996280

Data are presented as mean ± SE. Different lowercase superscript letters indicate notable differences at *p* < 0.05. “BJS-1-3” means BJS-1-3 fermentation-broth-treated poplar group; “CK” means sterilized fermentation-broth-treated poplar group.

## Data Availability

Most data are presented in the article and SRA database (Bioproject accession number: PRJNA913794). The raw data are presented in supplementary material. The other materials can be provided upon request.
